# Targeting neurotensin as a potential novel approach for the treatment of autism

**DOI:** 10.1186/1742-2094-7-58

**Published:** 2010-10-01

**Authors:** Ahmad Ghanizadeh

**Affiliations:** 1 Research Center for Psychiatry and Behavioral Sciences, Shiraz University of Medical Sciences, Hafez Hospital, Shiraz, Iran; 2 Department of Psychiatry, Shiraz University of Medical Sciences, Hafez Hospital, Shiraz, Iran

## Abstract

The pathophysiology of autism remains obscure. Recently, serum neurotensin levels in children with autistic disorder have been found to be higher than those of normal children. Neurotensin is known to intensify neuronal NMDA-mediated glutamate signaling, which may cause apoptosis in autism. Further, an imbalance of glutamate/GABAergic system in autism has been described. These observations lead to a postulate that neurotensin may accentuate the hyperglutaminergic state in autism, leading to apoptosis. Targeting neurotensin might be a possible novel approach for the treatment of autism.

## 

A recently published study in Journal of Neuroinflammation reports the finding that neurotensin (NT) is elevated in the serum of young children with autistic disorder [1]. In cultured rat cortical neurons, NT has been shown to increase glutamate outflow and to intensify N-methyl D-aspartic acid- (NMDA-) mediated glutamate signaling [2]. In addition, NT may enhance glutamate transmission and, in particular, activate NMDA receptors [3,4]. Such overstimulation of NMDA glutamate receptors can lead to excitotoxicity [5]. Thus, factors that modulate glutamatergic transmission may affect glutamate-induced cell apoptosis.

Previous studies have suggested a possible role for a hyperglutaminergic state in autism [6]. Further, an antagonist of the NMDA glutamate receptor, memantine, has been shown to improve some symptoms in autism [7]. In contrast, there have been conflicting reports regarding the effects of NT on GABAergic synapses. At least one study has reported that NT inhibits GABAergic synaptic transmission in rats [8]. Other studies have indicated that NT enhances GABA release [9], activates GABAergic interneurons in rat prefrontal cortex [10], and increases GABAergic activity in rat hippocampus [11]. NT may act in the CNS as an atypical neuroleptic [12]. Studies using an antagonist of the NT receptor subtype 1 (NTS1) have elucidated the functions driven by this receptor [13], and antagonism of NTS1 has been suggested as a novel therapeutic approach for the treatment of Parkinson's disease [4].

The higher serum levels of NT in young patients with autistic disorder does not necessarily indicate a casual role in autism [1]. Elevated NT levels in autistic disorder could be a result of inflammation. However, considering the known imbalance in glutamate-to-GABA ratios in children with autism [14], the higher levels of glutamate in autism [14], the downregulation of GABA(A) receptors in autism [15], and the role of NT in excessive activation of the NMDA receptor and apoptosis [3,4], NT may mediate brain damage in addition to activating inflammatory processes in autism. These observations collectively suggest a hypothesis that modulation of NT or of its receptors, in combination with traditional drugs, may provide a novel approach for the management of autism.

## Competing interests

The author declares that they have no competing interests.
